# Filamentous phages of *Ralstonia solanacearum*: double-edged swords for pathogenic bacteria

**DOI:** 10.3389/fmicb.2013.00325

**Published:** 2013-11-04

**Authors:** Takashi Yamada

**Affiliations:** Department of Molecular Biotechnology, Graduate School of Advanced Sciences of Matter, Hiroshima UniversityHigashi-Hiroshima, Japan

**Keywords:** filamentous phage, integration, phytopathogen, *Ralstonia solanacearum*, virulence change

## Abstract

Some phages from genus *Inovirus* use host or bacteriophage-encoded site-specific integrases or recombinases establish a prophage state. During integration or excision, a superinfective form can be produced. The three states (free, prophage, and superinfective) of such phages exert different effects on host bacterial phenotypes. In *Ralstonia solanacearum*, the causative agent of bacterial wilt disease of crops, the bacterial virulence can be positively or negatively affected by filamentous phages, depending on their state. The presence or absence of a repressor gene in the phage genome may be responsible for the host phenotypic differences (virulent or avirulent) caused by phage infection. This strategy of virulence control may be widespread among filamentous phages that infect pathogenic bacteria of plants.

## FILAMENTOUS PHAGES AND PATHOGENIC BACTERIA

Bacteriophages belonging to the genus *Inovirus* are filamentous particles containing a circular single-stranded (ss) DNA genome. Infection with this kind of phage does not cause host cell lysis, but establishes a persistent association between the host and phage, producing and releasing phage particles from the growing and dividing host cells. In general, the genome of inoviruses, represented by *Escherichia coli* F-pillus specific phage Ff (f1, fd or M13), is organized in a modular structure, in which functionally related genes are grouped together (Horiuchi et al., 2009; Rakonjac et al., 2011). Three functional modules are always present: the replication module, the structural module, and the assembly and secretion module. The replication module contains the genes encoding rolling-circle DNA replication and single-strand DNA (ssDNA) binding proteins *gII*, *gV*, and *gX* (Horiuchi et al., 2009). The structural module contains genes for the major (*gVIII*) and minor coat proteins (*gIII*, *gVI*, *gVII*, and *gIX*), and gene *gIII* encodes the host recognition or adsorption protein pIII (Wang et al., 2006). The assembly and secretion module contains the genes for morphogenesis and extrusion of the phage particles (*gI* and *gIV*; Marvin, 1998). Gene *gIV* encodes protein pIV, an aqueous channel (secretin) in the outer membrane, through which phage particles exit from the host cells (Marciano et al., 1999). Some phages encode their own secretins, whereas others use host products (Davis et al., 2000).

Because inoviruses coexist with their host cells, infection by these phages can mediate conversion of the host bacterial phenotypes in various ways. In pathogenic bacteria of either animals or plants, virulence is frequently affected by phage infection. For example, infection of *Xanthomonas campestris *pv. *oryzae *NP5850 by the filamentous phages Xf and Xf2 enhanced virulence, possibly because of overproduction of extracellular polysaccharides (EPS) by the phage-infected bacterial cells (Kamiunten and Wakimoto, 1982). Tseng et al. (1990) also reported that infection of *X*. *campestris *pv. *campestris *by the filamentous phage Lf increased virulence by promoting EPS production. Filamentous phages are assembled at the host cell surface and secreted into the environment. However, once then cells form colonies on the semi-solid medium (and possibly within the liquid medium), some fractions of secreted phage population are bound to stay trapped in the colony, potentially accumulating to high concentrations and forming a matrix surrounding the cells in the colony. These trapped phage particles may serve to cross-link cells to give high densities and induce biofilms. This situation was reported for small colony variant formation in *Pseudomonas aeruginosa* depending on phage Pf4 activity (Webb et al., 2004; Rice et al., 2009). More direct involvement of filamentous phages in host virulence is well characterized in *Vibrio cholerae.* The pathogenicity of this severe diarrheal disease-causing bacterium depends on two key virulence factors, the toxin co-regulated pilus (TCP) and cholera toxin. Cholera toxin genes are encoded on the filamentous phage CTXϕ and introduced into bacterial cells by phage integration mediated by the host *dif*/XerCD recombinase system (Huber and Waldor, 2002; Davis and Waldor, 2003). In *Ralstonia solanacearum*, infection by ϕRSS1 induced the early expression of *phcA*, a global virulence regulator, and also enhanced twitching motility (Addy et al., 2012b).

Contrasting with these virulence-enhancing effects of ϕRSS1, loss of virulence was also reported in *R. solanacearum*. *R. solanacearum* completely lost virulence through infection with two other filamentous phages ϕRSM1 and ϕRSM3 (Addy et al., 2012a). Many virulence factors were significantly reduced in ϕRSM-infected cells. These opposing effects of different filamentous phages on *R. solanacearum* virulence makes it an ideal study model system for understanding the effect of filamentous phage on their hosts. Here I will describe the role of filamentous phage in the virulence of *R. solanacearum* and suggest a causative relationship between a phage-encoded transcriptional repressor and *R. solanacearum* pathogenicity.

## *Ralstonia Solanacearum* AND BACTERIAL WILT

*Ralstonia solanacearum *is a Gram-negative β-proteobacterium that causes bacterial wilt disease in many important crops including tomato, potato, tobacco, and eggplant. Because of its wide geographic distribution and unusually broad host range (more than 50 plant families), it is responsible for significant crop losses worldwide (Hayward, 2000). Once the bacteria enter a susceptible host, they colonize the intercellular spaces of the root cortex and vascular parenchyma. The bacteria eventually enter the xylem and spread into the upper parts of the plant, causing wilt (Vasse et al., 2000; Kang et al., 2002; Yao and Allen, 2007). The development of bacterial wilt disease depends on bacterial pathogenicity and virulence (Carney and Denny, 1990; Denny, 2006). *R. solanacearum *virulence is additive, complex, and involves the production of multiple virulence factors (Schell, 2000; Genin and Boucher, 2002). For example, exopolysaccharide I (EPSI), a large nitrogen-rich acidic exopolysaccharide (Lavie et al., 2002), is thought to be an important virulence factor. It enhances the speed and extent of stem infection spreading from the root (Saile et al., 1997) and is presumed to cause wilting by restricting water flow through xylem vessels (Garg et al., 2000). In addition to EPSI, *R. solanacearum *secretes enzymes that degrade the plant cell wall through the type II secretion system (T2SS). Pectinolytic enzymes fragment pectin into oligomers, which act as a substrate for bacterial growth (Tans-Kersten et al., 2001). The breakdown of pectin enhances virulence by facilitating bacterial movement through pectin-rich regions such as vascular bundles (Gonzalez and Allen, 2003). Cellulolytic enzymes also facilitate bacterial invasion of roots and/or penetration of xylem vessels by degrading cellulosic glucans in the cell wall (Liu et al., 2005). In addition to T2SS-mediated secreted proteins, the type IV pilus (Tfp) is believed to be another virulence factor of *R. solanacearum* (Davis and Waldor, 2003). This protein forms a surface appendage that is responsible for twitching motility and polar attachment to host cells or to plant roots, and enhances the severity of wilt disease (Liu et al., 2001; Kang et al., 2002).

Expression of the pathogenesis and virulence genes in *R. solanacearum* is controlled by a complex regulatory network (Schell, 2000; Genin and Boucher, 2002; Denny, 2006) and is drastically affected by various environmental factors. The regulation is outlined as follows: the transcriptional regulator PhcA plays a critical role in the regulatory network. Abundant PhcA activates production of multiple virulence factors such as EPSI and cell wall degrading enzymes (CWDE). PhcA is activated by a quorum sensing system mediated by the two-component regulatory system PhcS/PhcR that responds to thereshold levels of 3-OH palmitic acid methylester (3-OH PAME), an autoinducer of quorum sensing that controls virulence. Therefore, the levels of 3-OH PAME, cell density, as well as cell surface nature all affect virulence in *R. solanacearum*.

## THREE STATES OF FILAMENTOUS PHAGE ϕRSS WITH DIFFERENT EFFECTS ON HOST VIRULENCE

**FIGURE 1:**
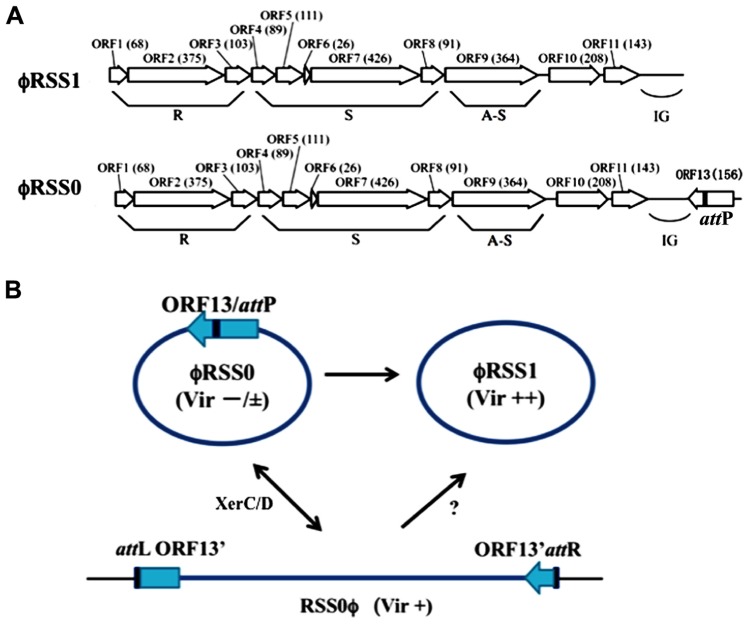
**ϕRSS type filamentous phages infecting *R. solanacearum.*** **(A)** Genomic organization of ϕRSS1 and ϕRSS0 (Kawasaki et al., 2007; Yamada, 2011) shown in a linear form. ORFs or genes are represented by arrows oriented in the direction of transcription. The functional modules for replication (R), structure (S), and assembly secretion (A-S) are indicated according to the M13 model (Marvin, 1998). The region containing the *att*P sequence is also indicated. **(B)** Interrelationship between three states of ϕRSS phages. The phage genomic DNA is shown in a circular form where most genes are not shown. ϕRSS0 is equipped with a 626-nt element containing ORF13, within which *att*P (*dif*) is located. This element is missing in ϕRSS1. The processes of interconversion between ϕRSS0 and ϕRSS1 are not known. ϕRSS0 is integrated at the *dif* site (*att*B) on the host genome. The prophage state is shown as RSS0ϕ, where the left and right borders are indicated as *att*L and *att*R, respectively. This integration (reversible) is mediated by the host XerCD system. ϕRSS1 may be produced directly from RSS0ϕ. The three states of ϕRSS0-type phage (ϕRSS0, ϕRSS1, and ϕRSS0 prophage) affect host *R. solanacearum* cells differently after infection, especially in host virulence. Compared with wild-type virulence (+), ϕRSS1 enhances (++) and ϕRSS0 reduces (-/±) the host virulence.

ϕRSS1 was isolated from a soil sample collected from tomato crop fields (Yamada et al., 2007). ϕRSS1 particles have a flexible filamentous shape 1,100 ± 100 nm in length and 10 ± 0.5 nm in width, giving a morphology resembling coliphage Ff (M13, f1 or fd; Buchen-Osmond, 2003; ICTVdB). The ϕRSS1 particles contain a ssDNA genome (6,662 nt; DDBJ accession no. AB259124), with a GC content of 62.6%. There are 11 open reading frames (ORFs), located on the same strand (**Figure 1A**). The ϕRSS1 gene arrangement is consistent with the general arrangement of Ff phages. Genomic Southern blot hybridization showed several examples of ϕRSS1-related sequences integrated in the genomes of various *R. solanacearum* strains (Yamada et al., 2007). A ϕRSS1-related phage (designated ϕRSS0) was induced and isolated from one such cross-hybridizing strain (C319) by infection with another phage (jumbo phage ϕRSL1). The DNA sequence of ϕRSS0 was very similar to ϕRSS1, but contained an extra 626 nt at ϕRSS1 position 6,628, next to the intergenic region (IG), giving an entire genomic size of 7,288 nt (GenBank accession no. JQ408219). Within the ϕRSS0 extra region, an ORF (ORF13) of 468 nt, corresponding to 156 amino acid residues, in a reversed orientation compared with the other ORFs, was found (**Figure 1A**). The amino acid sequence of ORF13 showed similarity to DNA-binding phage transcriptional regulators (accession no. B5SCX5, E-value = 1e-29).

Using inverse PCR with the new phage nucleotide sequences as primers, the prophage (ϕRSS0)-junctions (*att*L and *att*R) in strain C319 were obtained and their nucleotide sequences determined. It was found that both *att*L and *att*R contained repeated elements, corresponding to the *dif* sequence of *R. solanacearum* GMI1000 (Carnoy and Roten, 2009). This repeated sequence, 5′-TATTT AACAT AAGAT AAAT-3′, was also found at the 3′ end of ORF13 on the RSS0 genome, suggesting that it serves as *att*P.

Taken together, these results indicated that ϕRSS1 (with a genome size of 6,662 nt) is a truncated form of the larger phage ϕRSS0 (with a genome size of 7,288 nt). The 626 nt ϕRSS0 sequence missing from ϕRSS1 contains *att*P (corresponding to the *dif* sequence) and ORF13, a possible regulatory gene (Yamada, 2011; Tasaka et al., unpublished). ϕRSS0 is integrated at the *dif* site, similar to CTXϕ of *V. cholerae*, which uses the host XerCD recombination system (Huber and Waldor, 2002). ORF13 encoded on ϕRSS0 may function as a phage immunity factor, because strain C319 (ϕRSS0 lysogen) is resistant to secondary infection by ϕRSS0. C319 is susceptible to ϕRSS1, thus ϕRSS1 (without ORF13) may be an escaped superinfective phage. These three states of ϕRSS phages and their interrelationships are shown in **Figure 1B**.

Upon infection by the ϕRSS1 phage, the host *R. solanacearum* cells showed several abnormal behaviors, including less turbidity and frequent aggregation in the liquid culture, less coloration of colonies on plates, and a decreased growth rate (approximately 60% of the normal rate). More interestingly, ϕRSS1-infected cells showed enhanced virulence on tobacco (Yamada et al., 2007) and tomato plants (Addy et al., 2012b). In the case of strain C319 (ϕRSS0 lysogenic), inoculated tobacco plants showed wilting symptoms of grade 2–3 at 14 days post-inoculation (p.i.), whereas tobacco plants inoculated with ϕRSS1-infected C319 cells wilted earlier; grade 2–3 symptoms were observed at 10 days p.i. and plants were almost dead at 14 days p.i. (Yamada et al., 2007). Effects on host virulence by infection with ϕRSS0 in its free form (not prophage) were also examined. To make wilting symptoms clear, tomato-tropic *R. solanacearum* strain (MAFF 106603) in tomato experimental system was used. The cells were infected with either ϕRSS0 (free) or ϕRSS1. The physiological features of ϕRSS0-infected *R. solanacearum* MAFF 106603 cells were almost the same as ϕRSS1-infected MAFF 106603 cells, except that the ϕRSS0-infected cells formed colonies of more mucoid appearance on CPG plates. When MAFF 106603 (wild-type) cells were inoculated into the major stem of tomato plants, all 12 plants showed wilting symptoms as early as 3 days p.i. and died 5–7 days p.i. ϕRSS1-infected cells of MAFF 106603 inoculated into tomato in the same way caused wilting earlier, at 2 days p.i., and all 12 plants died by 5 days p.i. In contrast, tomato plants inoculated with ϕRSS0-infected cells showed wilting symptoms much later: most plants (10 of 12) survived after 7 days and a few plants did not show any symptoms until 23 days p.i. Therefore, ϕRSS0 infection caused reduced virulence in host bacterial cells (Tasaka et al., unpublished). The virulence-enhancing effects by ϕRSS1 infection can be explained as follows: surface-associated ϕRSS1 particles (or phage proteins) may change the surface nature (hydrophobicity) of host cells to generate a high local cell density, resulting in early activation of *phcA*, the global virulence regulator, or lack of *orf13*, which is absent from the ϕRSS1 genome (Addy et al., 2012b). The reduced virulence observed for ϕRSS0-infected cells may be caused by the function(s) of ORF13 encoded by ϕRSS0. These results are summarized in **Table 1**.

**Table 1:** Three states of filametous phages and their effects on host virulence.

Phage state	ϕ RSS-type	ϕRSM-type	Virulence
Free	ϕ RSS0	ϕ RSM3	+/- or -
Prophage	RSS0ϕ	RSM3ϕ	+
Superinfective mutant	ϕ RSS1	ϕ RSM3-ΔORF15	++

## EFFECT OF FILAMENTOUS PHAGE ϕRSM ON VIRULENCE OF *Ralstonia Solanacearum*

**FIGURE 2:**
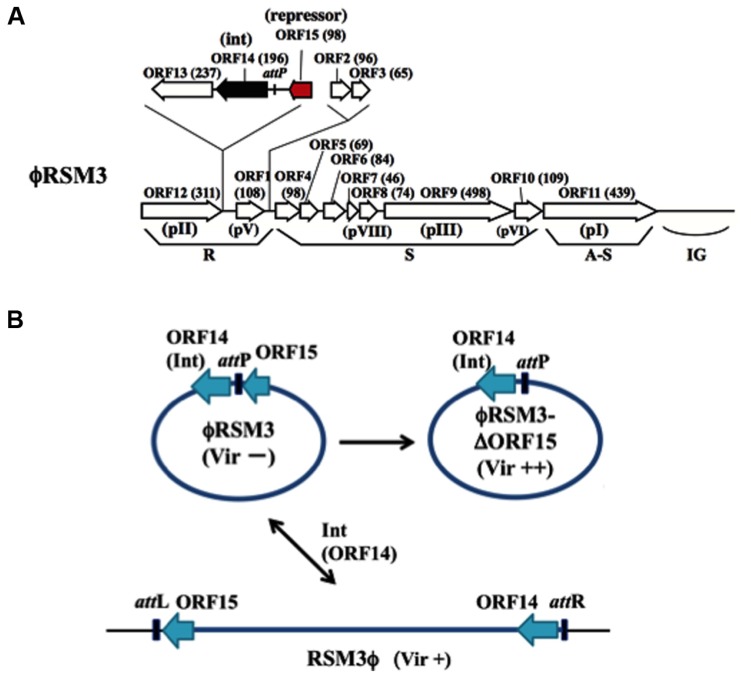
**ϕRSM type filamentous phages infecting *R. solanacearum.*** **(A)** Genomic organization of ϕRSM1 (Kawasaki et al., 2007; Askora et al., 2011). Map is drawn in the same way as in **Figure 1**. ORF14 and ORF15 correspond to Int (resolvase/invertase-like serine recombinase) and a newly identified DNA-binding repressor-like protein (Addy et al., 2012a), respectively. *att*P corresponds to a 13-nt 3′-end of the serine tRNA(UCG). **(B)** Interrelationship between three states of ϕRSM phages. For the experimental convenience ϕRSM3 was used in the study. The phage genomic DNA is shown in a circular form where most genes are not shown. ϕRSM3-δORF15 is a deletion mutant of ϕRSM3 lacking ORF15 (Addy et al., 2012a). ϕRSM3 is integrated at the tRNA(UCG) site (*att*B) on the host genome. The prophage state is shown as RSM3ϕ, where the left and right borders are indicated as *att*L and *att*R, respectively. This integration (reversible) is mediated by phage-encoded Int (ORF14). The three states of ϕRSM-type phage (ϕRSM3, ϕRSM3-δORF15, and RSM3ϕ prophage) affect host *R. solanacearum* cells differently after infection, especially in host virulence. Compared with wild-type virulence (+), ϕRSM3-δORF15 enhances (++) and ϕRSM3 suppress (-) the host virulence.

ϕRSM1 is also a soil-isolated filamentous phage 1,400 ± 300 μm long and 10 ± 0.7 nm wide (Yamada et al., 2007). The infection cycle of ϕRSM1 phage resembles that of ϕRSS1. The genome of ϕRSM1 is 9,004 nt long (DDBJ accession No. AB259123) with a GC content of 59.9%. There are 12 putative ORFs located on the same strand and three on the opposite strand. The ϕRSM1 genes are shown in **Figure 2A**, in comparison with the conserved gene arrangement of M13-like phages (Kawasaki et al., 2007). Here, ORF13, ORF14, and ORF15 (reversely oriented) are inserted between ORF12, corresponding to pII as a replication protein, and ORF1, corresponding to a ssDNA-binding protein like pV, in the putative replication module. ORF13, ORF14, and ORF15 show amino acid sequence similarity to a proline-rich transmembrane protein, a resolvase/DNA invertase-like recombinase, and a putative phage repressor, respectively (Kawasaki et al., 2007; Addy et al., 2012a). There are two additional ORFs (ORF2 and ORF3) between the replication and structural modules. The functions of these ORF-encoded proteins are not known. In genomic Southern blot hybridization, two different types of ϕRSM1-related prophage sequences were detected in *R. solanacearum* strains. Strains of type A include MAFF211270 and produce ϕRSM1 itself, and strains of type B (giving different restriction patterns) are resistant to ϕRSM1 infection, but are susceptible to ϕRSM3 (see below). By determining the nucleotide sequences of junction regions of the ϕRSM1-prophage in the MAFF 211270 chromosomal DNA, an *att*P/*att*B core sequence was identified as 5′-TGGCGGAGAGGGT-3′, corresponding to positions 8,544–8,556 of ϕRSM1 DNA, located between ORF14 and ORF15. Its nucleotide sequence is identical to the 3′-end of the host *R. solanacearum* gene for serine tRNA(UCG) in the reverse orientation. A ϕRSM1-like prophage (type B) in strain MAFF 730139, designated ϕRSM3, was obtained by PCR amplification using appropriate primers containing these *att* sequences (Askora et al., 2009). Compared with the ϕRSM1 genome, the ϕRSM3 prophage sequence (8,929 nt) is 75 nt shorter. The sequences show 93% nucleotide identity and major differences are found within two regions; positions 400–600 and positions 2,500–3,000 in the ϕRSM1 sequence. The former region corresponds to ORF2, which is inserted between the replication module (R) and the structural module (S), and has no similarity between the two phages. The latter falls into the possible D2 domain of ORF9 (pIII), which determines the host range. All other ORFs identified along the ϕRSM3 are highly conserved between two phages (over 90% amino acid identity). It is interesting that the amino acid sequence of ORF14 (putative DNA invertase/recombinase) is 100% identical in the two phages. The gene arrangement of ϕRSM3, which is almost the same as ϕRSM1, is shown in **Figure 2A**.

As described above, the genomes of ϕRSM phages are sometimes integrated in the host genome. Askora et al. (2011) demonstrated that the integration is mediated by the phage-encoded recombinase (ORF14 of ϕRSM1/ϕRSM3), which has significant homology to resolvases/DNA invertases (small serine recombinases), with *att*P/*att*B corresponding to the 3′ end of the host serine tRNA(UCG) gene in the reverse orientation. This is the first case of filamentous phages demonstrated to integrate into the host genome by its endogenously encoded integrase (Askora et al., 2012). The same unit of integration (ϕRSM Int/*att*P) was found in a *Ralstonia pickettii* 12J phage and in *Burkholderia pseudomallei* 668 prophages (Askora et al., 2012). Together with these phages, it would not be surprising if similar Int-containing filamentous phages occur widely in nature.

Infection by ϕRSM1 or ϕRSM3 establishes a persistent association between the host and the phage. Upon infection by ϕRSM phages, the host cells showed some abnormal behaviors and characteristics, such as frequent aggregation, dark coloration, and relatively small colony size, as observed in ϕRSS infection. When cells of MAFF 106611 (ϕRSM3 lysogenic strain) or MAFF 106603 (not lysogenic) were inoculated into tomato plants, all 20 plants showed wilting symptoms as early as 3 days p.i., whereas none of the 20 tomato plants inoculated with free-ϕRSM3-infected cells (for example, MAFF 106603) showed any wilting symptoms until 4 weeks p.i. (Addy et al., 2012a). This loss of virulence effect of ϕRSM3 infection can be explained in three ways: (i) reduced twitching motility and reduced amounts of type IV pili (Tfp), (ii) lower levels of β-1,4-endoglucanase (Egl) activity and EPS production, and (iii) reduced expression of certain virulence/pathogenicity genes (*egl*, *pehC*, *phcA*, *phcB*, *pilT,* and *hrpB*) in the infected cells (Addy et al., 2012a). This is supported restoring virulence in ϕRSM3 lysogen by deletion of ϕRSM3-encoded *orf15*, the gene for a putative repressor-like protein, was disrupted (Addy et al., 2012a). Therefore, ORF15 of ϕRSM3 may repress host genes involved in pathogenicity/virulence and consequently result in loss of virulence. With different strains as hosts, ϕRSM1 also gave similar results. The ϕRSM states and interaction with the host genome can be depicted similarly to ϕRSS phages, as shown in **Figure 2B**. These results are summarized and compared with the three states of ϕRSS in **Table 1**.

## PERSPECTIVE AND HYPOTHESIS

As seen here, for *R. solanacearum*, filamentous phages such as ϕRSS and ϕRSM are double-edged swords; sometimes they help bacteria to infect plants by enhancing bacterial virulence, and sometimes they interrupt bacterial infection of plants by repressing host genes involved in virulence. The contradictory effects of these phages may largely depend on the presence or absence of a phage-encoded regulatory protein. Two questions arise here: (i) How does the regulatory affect on the host genes; working alone, with other phage factors, or with host factors? (ii) How does such a regulatory gene become acquired by or lost from the phage genome? Concerning the first question, as shown in **Figure 1B**, attP is located within ORF13 on ϕRSS0 DNA, and after integration at attB on the host genome, a truncation of ORF13 (at the C-terminus) occurs. By creating a new stop codon in the reading frame, the size of ORF13 reduced from 156 to 130 aa with a 26-aa truncation at the C-terminus (Tasaka et al., manuscript in preparation). A DNA-biding motif (Helix-Turn-Helix) is located in the N-terminal moiety and the C-terminal region may have some regulatory function (such as ligand-binding). This suggests a functional difference of the ORF13 protein before and after integration. One possibility is that the full length ORF13 (ORF15 in ϕRSM phages) expressed from free phages may function to preferentially regulate host genes and the truncated (or modified) form expressed from the prophage may function to stabilize the prophage state and phage immunity, protecting against infections by related phages (Hypothesis 1). This hypothesis is compatible with the observation that once a ϕRSS and ϕRSM prophage state was established, the phage genomic DNA and phage particles seldom appeared in the lysogenic strains. Like ϕRSM, the DNA or the phage particles are not identified in the lysogen, even though the orf15 encoding the putative repressor ORF15 is not changed before and after the host integration. Because ORF14 integrase (serine recombinase) of ϕRSM phages likely mediates both integrative and excessive recombinations (Askora et al., 2011), some additional factors are required to mediate prophage replication or excision. The function, regulatory mechanism, and effect on virulence of ϕRSS orf13 or ϕRSM orf15 remain to be investigated by direct expression of the corresponding gene in an appropriate host strain. In our preliminary trial where the coding region of ORF15 of ϕRSM3 (ORF13 of ϕRSS0) was expressed from a plasmid under the control of lacP and introduced into appropriate host strains, no transformants with a correct construct appeared (colonies that appeared on the selection plates after transformation all contained deleted inserts). One of the explanation for this is putative toxic effect of ORF13 and ORF15 on the host when expressed under these conditions. Some additional factors encoded on the phage genome may be involved in the appropriate regulation, interacting with ORF13 or ORF15 (Hypothesis 2). Further studies with mutated constructs of ORF13 or ORF15 are required to test these hypotheses.

As for question of loss of a repressor protein, a 626-nt sequence unit containing *orf13* and *att*P detected in ϕRSS0 and missing from ϕRSS1 plays a crucial role in ϕRSS dynamics. The origin of such a sequence and the mechanism how it comes in or out of the phage are largely unknown. However, the possibility of two forms from a phage is important. Apparently, ϕRSS1-infected bacterial cells have an advantage in the pathogenic lifestyle. Nevertheless, the virulence is not always necessary for this soil-borne bacterium. Infection of ϕRSS0 provides the host cells with a sophisticated mechanism to control their virulence. Similar mechanisms may function in other pathogenic bacteria (Hypothesis 3). To test this hypothesis, various systems involving pathogenic bacteria and their filamentous phages should be examined. For example, ϕRSS1-like superinfective phage Cf1tv spontaneously appeared from the Cf1t lysogenic strain of *Xanthomonas campestris* pv. *citri *(Kuo et al., 1994). Unfortunately, nucleotide sequence information is not available for this phage. Similar kinds of phage involvement in host virulence regulation may be universal, because ϕRSS- or ϕRSM-related sequences are frequently found in various bacterial genomic sequences in the databases, including *R. pickettii* (CP001645), *Ralstonia syzygii* (FR854090), *Burkholderia rhizoxinica* (FR687359), *Pectobacterium wasabiae* (CP001790), and *Erwinia carotovora* (BX950851). There are also other filamentous phages that have lysogenic cycles, including *X.* *campestris *phages Cf1c (Kuo et al., 1991), Cf1t (Kuo et al., 1987a,b), Cf16v1 (Dai et al., 1980), and ϕLf (Lin et al., 2000); *Xylella fastidiosa* phage Xfϕ-f1 (Simpson et al., 2000); *Yersinia pestis *phages CUSϕ-2 (Gonzalez and Allen, 2003) and Ypfϕ (Derbise et al., 2007); Nf of *Neisseria meningitidis* (Kawai et al., 2005), and *V. cholerae *phages VGJϕ (Campos et al., 2003) and VCYϕ (Xue et al., 2012). The host bacteria of these phages are plant or animal pathogens.

## Conflict of Interest Statement

The authors declare that the research was conducted in the absence of any commercial or financial relationships that could be construed as a potential conflict of interest.
